# Emerging biofilm formation and disinfectant susceptibility of ESBL-producing *Klebsiella pneumoniae*

**DOI:** 10.1038/s41598-024-84149-x

**Published:** 2025-01-10

**Authors:** Hanan S. Khalefa, Amany A. Arafa, Dalia Hamza, Khaled A. Abd El-Razik, Zeinab Ahmed

**Affiliations:** 1 Department of Veterinary Hygiene and Management, Faculty of Veterinary Medicine, Cairo University, Giza, 12211 Egypt; 2 Department of Microbiology and Immunology, Veterinary Research Institute, National Research Centre, Dokki, Egypt; 3 Department of Zoonoses, Faculty of Veterinary Medicine, Cairo University, Giza, 12211 Egypt; 4 Department of Animal Reproduction, Veterinary Research Institute, National Research Centre, Dokki, Egypt

**Keywords:** *K. pneumonia*, Poultry, Wild bird, Equine, Human, ESBL, Biofilm genes, Microbiology, Biofilms

## Abstract

*Klebsiella pneumoniae* is an opportunistic pathogen responsible for various infections in humans and animals. It is known for its resistance to multiple antibiotics, particularly through the production of Extended-Spectrum Beta-Lactamases (ESBLs), and its ability to form biofilms that further complicate treatment. This study aimed to isolate and identify *K. pneumoniae* from animal and environmental samples and assess commercial disinfectants’ effectiveness against *K. pneumoniae* isolates exhibiting ESBL-mediated resistance and biofilm-forming ability in poultry and equine farms in Giza Governorate, Egypt. A total of 320 samples, including nasal swabs from equine (n = 60) and broiler chickens (n = 90), environmental samples (n = 140), and human hand swabs (n = 30), were collected. *K. pneumoniae* was isolated using lactose broth enrichment and MacConkey agar, with molecular confirmation via PCR targeting the *gyrA* and *magA* genes. PCR also identified ESBL genes (*bla*_TEM_*, bla*_SHV_*, bla*_CTX-M_*, bla*_OXA-1_) and biofilm genes (*luxS, Uge, mrkD*). Antimicrobial susceptibility was assessed, and the efficacy of five commercial disinfectants was evaluated by measuring inhibition zones. *Klebsiella pneumoniae* was isolated from poultry (13.3%), equine (8.3%), wild birds (15%), water (10%), feed (2%), and human hand swabs (6.6%). ESBL and biofilm genes were detected in the majority of the isolates, with significant phenotypic resistance to multiple antibiotics. The disinfectants containing peracetic acid and hydrogen peroxide were the most effective, producing the largest inhibition zones, while disinfectants based on sodium hypochlorite and isopropanol showed lower efficacy. Statistical analysis revealed significant differences in the effectiveness of disinfectants against *K. pneumoniae* isolates across various sample origins (P < 0.05). The presence of *K. pneumoniae* in animal and environmental sources, along with the high prevalence of ESBL-mediated resistance and biofilm-associated virulence genes, underscores the zoonotic potential of this pathogen. The study demonstrated that disinfectants containing peracetic acid and hydrogen peroxide are highly effective against ESBL-producing *K. pneumoniae*. Implementing appropriate biosecurity measures, including the use of effective disinfectants, is essential for controlling the spread of resistant pathogens in farm environments.

## Introduction

*Klebsiella pneumoniae* is a significant pathogen affecting both animal and human health. In humans, *K. pneumoniae* is a leading bacteria responsible for nosocomial infections, contributing to serious conditions such as pneumonia^1^. It can lead to severe respiratory issues and increased mortality in poultry, especially in crowded environments^2^. Additionally, in equines, this bacterium can cause respiratory symptoms like hemorrhagic nasal discharge and pneumonia, and it has also been linked to abortion in pregnant mares^3,4^.

*K. pneumoniae* is notorious for acquiring beta-lactam resistance genes, including extended-spectrum beta-lactamases (ESBLs), which pose serious treatment challenges and increase healthcare costs^5,6^. These strains resist a broad range of beta-lactam antibiotics, including penicillins and cephalosporins. ESBL-producing *K. pneumoniae* strains present a particularly significant challenge due to their resistance mechanisms and potential for horizontal gene transfer^7^.

Wild birds serve as reservoirs, shedding the bacterium into the environment, which can lead to contamination of water sources and subsequent transmission to humans and animals^8,9^. *K. pneumoniae* contamination in water sources poses significant public health risks, particularly through pathways such as agricultural runoff, sewage discharge, and animal feces^10,11^. Contaminated water can transmit the bacterium to humans and animals, especially in vulnerable populations lacking adequate sanitation. Several studies have suggested that microbes form biofilms in poultry water systems^12,13^. Notably, *K. pneumoniae* strains in water may carry antibiotic resistance and virulence genes, posing a risk of spreading these traits to other pathogens^14^.

Biofilms are bacterial communities composed of one or more species embedded within an extracellular matrix made of polysaccharides, proteins, and DNA. Their formation enhances the bacterium’s resistance to antimicrobial agents and external stressors, playing a pivotal role in its pathogenicity^15^. For *K. pneumoniae*, biofilms serve as a key virulence mechanism, offering increased environmental resilience and acting as reservoirs for genetic exchange, thereby promoting the spread of antimicrobial resistance^16^.

Studies have identified numerous genes associated with biofilm formation, including those involved in the production of aerobactin *(iutA*), allantoin (*allS*), type I fimbriae (*fimA* and *fimH*), type III fimbriae (*mrkA* and *mrkD*), capsular polysaccharides (CPS) (*cpsD, treC, wabG, wcaG, wzc, k2A*, and *wzyK2*), quorum sensing (QS) (*luxS*), and colonic acid (*wcaJ*)^17–19^. Moreover, the *uge* gene, which encodes UDP-galacturonate 4-epimerase crucial for LPS biosynthesis, supports biofilm formation by maintaining cell surface integrity, aiding initial adhesion, and enhancing protection within the biofilm^20,21^.

Recent evidence indicates that biofilm formation is significantly more prevalent in ESBL-producing *K. pneumoniae* strains, suggesting that therapeutic guidelines should incorporate the impact of biofilm production when optimizing treatment strategies and clinical outcomes, rather than relying solely on antibiotic susceptibility test results, as these biofilms can protect bacteria from both antimicrobial agents and immune defenses^22,23^.

The genetic diversity among clinical isolates of *K. pneumoniae*, including variations in virulence and resistance genes, underscores the challenge of managing infections caused by this pathogen^14,24^. Understanding the mechanisms behind genetic diversity in *K. pneumoniae*, including specific virulence and resistance genes, their interactions, and their contributions to the pathogen’s overall virulence, is crucial for developing effective strategies to combat biofilm-associated infections, improve patient outcomes, and ultimately lead to new therapeutic approaches for managing and treating these infections^25,26^.

Biocides have long been used to reduce microbial populations on various surfaces, serving as a key tool in preventing the proliferation of multidrug-resistant (MDR) organisms and the transmission of infections^27^. According to the European Commission, a “biocidal product” is defined as a substance that contains one or more active ingredients or facilitates their formation, and is designed to "destroy, deter, or render harmless" microorganisms through non-physical or non-mechanical means to mitigate or eliminate potential adverse effects on host health^28^. These compounds are typically categorized into four groups based on their primary characteristics and mode of action: antiseptics, sterilants, disinfectants, and preservatives^29,30^. A lack of adequate information regarding sanitation programs hinders effective regulation and control of zoonotic disease spread, thereby increasing health risks for both animals and farmers^31^. Farms implement sanitary measures such as spraying disinfectants in sheds and removing feces^32^.

According to Stringfellow et al.^32^ the farms implemented sanitation protocols, including disinfectants in shelters and removing feces. Disinfectants are frequently implemented according to the manufacturing guide. Nevertheless, the efficacy of disinfectants can be influenced by various factors, including the degree of organic burden, humidity, temperature, dilution rate, pH, and water hardness. Furthermore, the use of disinfectants without adequate validation and evaluation can lead to a substantial increase in selective pressure, which in turn results in a progressive decrease in the sensitivity of organisms to the disinfectants used. This can result in cross-resistance, in which the organisms develop resistance to antibiotics that are significant to public health^33^.

Despite the recognized role of biofilm formation in *K. pneumoniae* virulence and spread, there is a notable gap in understanding the prevalence of ESBL-producing *K. pneumoniae* and its association with biofilm-related genes in livestock, particularly poultry, and equine. This study aims to characterize *K. pneumoniae* in livestock, their environments, and workers, focusing on biofilm formation, antibiotic resistance, ESBL genes, and genetic diversity. Additionally, it evaluates the effectiveness of five disinfectants as biosecurity measures to reduce infection risks in humans and animals.

## Materials and methods

### Sample collection and preparation

A total of 320 samples were collected between June 2022 and April 2023 from eight farms in Giza Governorate, Egypt, where poultry and equines were affected by respiratory infections. These included 150 nasal swabs from animals with respiratory illness (60 from equines and 90 from broiler chickens), 140 environmental samples, and 30 hand swabs from farm workers. Among the farms, three were poultry farms, and five were equine farms. All samples were analyzed for the presence of *K. pneumoniae*.

The nasal and hand swabs were collected using sterile swabs, with nasal samples placed in 2 mL of sterile saline (0.9% NaCl) and hand swabs in 9 mL of sterile buffered peptone water (Oxoid, Basingstoke, Hampshire, England).

Environmental samples included 50 livestock drinking water samples (30 from equine water troughs and 20 from poultry drinking sources), 50 feed samples collected from animal pens, and 40 fecal samples from wild birds (10 crows, 10 pigeons, and 20 laughing doves).

Wild birds were captured using mist nets set up for six hours in farm areas and checked every 20 min to identify and mark birds to prevent resampling. Fresh fecal samples from the captured birds were collected using sterile cotton swabs and stored in 15-mL polyethylene tubes.

All samples, including drinking water collected in sterile 1-L glass bottles and feed samples in sterilized bags, were transported to the laboratory in an icebox at 4 °C for microbiological analysis.

### Isolation and identification of *K. Pneumoniae*

Each sample was inoculated into lactose broth (Oxoid, Hampshire, UK) and incubated overnight at 37 °C. Following enrichment, a loopful of the broth was streaked onto MacConkey agar (Oxoid, Hampshire, UK) and incubated at 37 °C for 18–24 h. To isolate *Klebsiella* species from the water samples, the membrane filtration technique was employed. For this process, 100 mL of each water sample was filtered through a sterile 0.45-µm pore-size membrane filter. The filters were then aseptically transferred onto the surface of MacConkey agar plates and incubated at 37 °C for 18–24 h.

Suspected *Klebsiella* spp. colonies were selected according to Collee et al.^34^ and subcultured to obtain pure cultures. To address the limitations of relying solely on colony morphology on MacConkey agar and to ensure accurate identification, the pure culture was subjected to a series of conventional biochemical tests, including catalase, oxidase, oxidative-fermentative, indole, methyl red, Voges-Proskauer, citrate, and triple sugar iron agar tests^35^. All reagents used for these biochemical tests were sourced from Oxoid (Oxoid, Hampshire, UK).

### Molecular confirmation of *K. Pneumoniae* isolates and detection of ESBL-mediated resistance and biofilm genes

#### Extraction of the genomic DNA

Two milliliters of enriched lactose broth were centrifuged at 13,000 rpm for 10 min. After discarding the supernatant, 200 µL of nuclease-free water was added to the pellet. The pellet was dissolved and heated in a water bath at 100 °C for 10 min, then cooled to 20 °C and left overnight. The mixture was centrifuged again at 13,000 rpm for 10 min, and the resulting supernatant was used as the DNA template for all PCR reactions^36^. The extracted DNA was stored at -20 °C for future use.

#### Molecular confirmation of *Klebsiella* spp. and *K. pneumoniae* isolates

*Klebsiella* isolates were confirmed by amplifying the *gyrA* gene specific to the *Klebsiella* genus, as previously described^37^. For the specific identification of *K. pneumoniae*, the *magA* primer was utilized^37^. Details of the primer sequences, amplicon sizes, and amplification conditions for both the *gyrA* gene and *magA* gene are provided in Table 1.

**Table 1:** Sequence of oligonucleotide primers used for PCR amplification of *Klebsiella* spp., ESBLs encoding genes and biofilm genes.

	Target gene	Primer sequence (5ʹ–3ʹ)	Amplicon size (bp)	Amplification conditions
*Klebsiella* spp.	*gyrA*	F: CGC GTA CTA TAC GCC ATG AAC GTA R: ACC GTT GAT CAC TTC GGT CAGG	441	Initial denaturation of 5 min at 94 °C followed by 35 cycles, denaturation at 94 °C for 30 s, annealing at 55 °C for 45 s, extension at 72 °C for 45 s, and final extension at 72 °C for 10 min
*K. pneumoniae*	*magA*	F: ATT TGA AGA GGT TGC AAA CGAT R: TTC ACT CTG AAG TTT TCT TGT GTTC	130	Initial denaturation of 5 min at 94 °C followed by 35 cycles, denaturation at 94 °C for 30 s, annealing at 55 °C for 30 s, extension at 72 °C for 40 s, and final extension at 72 °C for 10 min
ESBLs encoding genes	*bla*_TEM_	F: CGC CGC ATA CAC TAT TCT CAG AAT GA R: ACG CTC ACC GGC TCC AGA TTT AT	445	Initial denaturation of 5 min at 94 °C followed by 30 cycles, denaturation at 94 °C for 1 min, annealing at 61 °C for 1 min, extension at 72 °C for 1 min, and final extension at 72 °C for 5 min
	*bla*_SHV_	F: CTT TAT CGG CCC TCA CTCAA R: AGG TGC TCA TCA TGG GAA AG	237	
	*bla*_CTX-M_	F: ATG TGC AGY ACC AGTAAR GTK ATG GC R: TGG GTR AAR TAR GTS ACC AGA AYC AGC GG	593	
	*bla*_OXA-1_	F: ACA CAA TAC ATA TCA ACT TCG C R: AGT GTG TTT AGA ATG GTG ATC	813	
biofilm genes	*LuxS*	F: GCC GTT GTT AGA TAG TTT CACAG R: CAG TTC GTC GTT GCT GTT GATG	447	Initial denaturation of 2 min at 95 °C followed by 35 cycles, denaturation at 95 °C for 20 s, annealing at 53 °C for 30 s, extension at 72 °C for 45 s, and final extension at 72 °C for 7 min
	*Uge*	F: TCT TCA CGC CTT CCT TCA CT R: GAT CAT CCG GTC TCC CTG TA	535	
	*mrkD*	F: CCACCAACTATTCCCTCGAA R: ATGGAACCCACATCGACATT	226	

#### Molecular detection of ESBL encoding genes

All strains confirmed as *K. pneumoniae* were further screened for relevant ESBL-encoding resistance genes using multiplex PCR as previously described^38,39^. This PCR targeted *bla*_TEM_, *bla*_SHV_, *bla*_CTX-M_, and *bla*_OXA-1_ resistance determinant genes using specific oligonucleotide primer sets (Table 1). The PCR reaction was performed in a total volume of 25 µl, consisting of 3 µl of template DNA from each isolate, 12.5 µl of EmeraldAmp MAX PCR master mix (Takara, Japan), 0.5 µl of each primer (10 pmol/µl; Metabion, Germany), and PCR-grade water to complete the volume. Details of the primer sequences and amplification conditions are provided in Table 1.

All PCR products were electrophoresed on a 1.5% agarose gel (vivantis, Malysia) and visualized under a UV transilluminator. A 100 bp DNA ladder (Jenna Bioscience GmbH, Jenna, Germany) was run alongside the samples to determine the size of the PCR amplicon. A negative control, which included all PCR components except water instead of template DNA, was also included. The positive control consisted of the *E. coli* strain ATCC 25922.

#### Molecular detection of the biofilm genes

Uniplex PCR was performed on *K. pneumoniae* isolates to detect the biofilm genes (*luxS, Uge, and mrkD).* The reaction was performed according to Arafa and Kandil^22^. The primer sequences, amplification conditions, and amplicon sizes are presented in Table 1. Positive control was included in each set of reactions and the negative control was nuclease-free water that replaced the DNA sample in the PCR mix. The PCR products (S1–S3) were seen on 1.5% agarose gels.

##### Antimicrobial susceptibility test of selected ESBL-resistant *Klebsiella pneumoniae* isolates harboring biofilm-associated genes

Antimicrobial susceptibility testing was conducted on 18 selected *K. pneumoniae* isolates from various sources, exhibiting ESBL resistance and biofilm-associated genes, using the Kirby-Bauer disc diffusion method on Mueller–Hinton agar (Oxoid, Hampshire, UK). A total of 14 antimicrobial agents were tested, including gentamycin(GEN:10 µg), ampicillin (AMP: 10 µg), amoxicillin-clavulanic acid (AMC: 30 µg), erythromycin (ERY: 15 µg), Azithroyn (AZM: 30 µg), cefotaxime (CTX: 30 µg), Ceftazidime (CAZ:30 µg), nalidixic acid (NAL: 30 µg), ciprofloxacin (CIP: 5 µg), norfloxacin(NOR: 5 µg), chloramphenicol (CHL: 30 µg), tetracycline (TET: 30 µg), ceftriaxone (CT: 30 µg), and Clindamycin (CLN:2 µg ) (Oxoid, Hampshire, UK). The interpretation of inhibition zone diameters was based on the recommendations of the Clinical and Laboratory Standards Institute (CLSI)^40^**.** Isolates were classified as multidrug-resistant (MDR) if resistant to 3 different antimicrobial classes^41^**.**

### Effects of different disinfectants on ESBL-producing *Klebsiella pneumoniae* isolates harboring biofilm-associated genes

#### Selection of disinfectants

Five commercial disinfectants were prepared and utilized according to the manufacturer’s instructions at ready-to-use concentrations. Disinfectant A contained potassium peroxy-monosulfate and sodium hypochlorite with 1.1% active chlorine (Antec International TD, USA), disinfectant B comprised peracetic acid and hydrogen peroxide at 1.5% (Egy-Holland, Egypt), disinfectant C included a mix of 17.2% isopropanol and 0.28% ammonium compounds (IACs). Disinfectant D was formulated with quaternary ammonium compounds (Fluka Analytical, St. Louis, USA). Finally, Disinfectant E combined quaternary ammonium compounds, glutaraldehyde, formaldehyde, isopropanol, and pine oil (AdvaCare Pharma, USA). Each disinfectant was appropriately diluted per the manufacturer’s guidelines to achieve effective ready-to-use concentrations.

#### Efficacy testing of disinfectant

The Sixteen *Klebsiella pneumoniae* isolates selected from animal origin (Equine, poultry, and environment) were inoculated onto Petri plates containing brain–heart infusion agar (BHIA, France) to ensure their purity and vitality. A single colony from each isolate was subcultured on triple sugar iron agar (TSI, Italy) and incubated at 37 °C for 24 h, followed by an additional 24-h incubation at 36 °C ± 1 °C. The microorganisms were then suspended in a sterile saline solution to achieve a final concentration of 0.5 McFarland (1.5 × 10^8^ colony-forming units/ml) and evenly spread onto Mueller–Hinton agar (Oxoid, Hampshire, UK) using the rolling technique and sterile swabs. Six 7-mm wells were created in the agar: one in the center and five others approximately 20 mm away from the center, establishing the agar diffusion test setup. Each well was filled with 50 μL of a different disinfectant, and the plates were incubated overnight to determine the inhibition zones. The efficacy of each disinfectant was assessed by measuring the diameter of the microbial inhibition zones around the wells. Bacteria were considered sensitive if the inhibitory zone diameter exceeded 8 mm. The tests were conducted in triplicate, and the results were evaluated accordingly. A well containing 50 μL of sterile saline served as a negative control. All experiments were conducted using sterile techniques^42,43^.

### Statistical analyses

The data were analyzed using SPSS version 18.0, with a p-value of less than 0.05 considered statistically significant. Chi-square testing was used to compare multiple groups of categorical samples.

Isolates from various sources were grouped based on their antimicrobial resistance (AMR) traits using a heatmap with hierarchical clustering, generated with the “Heatmap” R package (version 4.2.2, R Foundation for Statistical Computing). Clustering of strains was performed using the pheatmap library (version 1.0.12)^44^.

## Results

### Occurrence of *K. Pneumoniae* isolates among different studied isolates

Out of 320 samples, 67 (20.9%) were positive for *Klebsiella* spp and 31 (9.7%) were positive for *K. pneumoniae*. Statistical analysis revealed significant variation among sources for the number of *Klebsiella* spp. positive samples (X^2^ = 12.171, P = 0.016).

From poultry, 90 samples were examined, with 30 (33.3%) positive for *Klebsiella* spp. and 12 (13.3%) for *K. pneumoniae.* Equine samples, totaling 60, had 9 (15%) positive for *Klebsiella* spp. and 8 (8.3%) for *K. pneumoniae*. Wild bird samples (40) showed 8 (20%) positive for *Klebsiella* spp. and 6 (15%) for *K. pneumoniae*. Water samples (50) yielded 12 (24%) positive for *Klebsiella* spp. and 5 (10%) for *K. pneumoniae*. Feed samples (50) had 4 (8%) positive for *Klebsiella* spp. and 1 (2%) for *K. pneumoniae*. Human hand swab samples (30) showed 4 (13.3%) positive for *Klebsiella* spp. and 2 (6.6%) for *K. pneumoniae*.

### Occurrence of ESBL-mediated resistance and biofilm genes in *K. Pneumoniae* isolates

The frequency and variation of ESBL-mediated resistance in *K. pneumoniae* isolates and biofilm-associated genes obtained from different animal sources and environments were displayed in the Table 2.

**Table 2:** Frequency of ESBL resistance genes and Biofilm-associated genes in *Klebsiella pneumoniae* isolates from Poultry, Equine, Human, and Environmental Samples.

Source	No. of examined samples	*Klebsiella* spp.	*K. pneumoniae*	ESBL encoding genes				*K. pneumoniae* virulence genes (biofilm genes)		
		No. positive* (%)	No. positive* (%)	*bla*_*TEM*_	*bla*_*SHV*_	*bla*_*CTX-M*_	*bla*_*OXA-1*_	*Uge*	*mrkD*	*luxS*
Poultry	90	30 (33.3)	12 (13.3)	12	12	11	2	8	7	6
Equine	60	9 (15)	5 (8.3)	4	4	4	0	4	4	4
Wild bird	40	8 (20)	6 (15)	6	6	6	1	3	3	3
Water	50	12 (24)	5 (10)	3	3	3	0	3	3	3
Feed	50	4 (8)	1 (2)	1	1	0	0	1	0	0
Human (hand’s swabs)	30	4 (13.3)	2 (6.6)	2	2	1	0	2	0	0
Total	320	67 (20.9)	31 (9.7)	28	28	24	3	21	17	16
*X* ^2^	–	12.171	4.650	3.644	1.296	1.241		1.194	1.098	1.458
*P* value	–	0.016	0.325	0.302	0.861	0.619	Nd	0.769	0.7775	0.691

*X*^2^: Chi-Square.

*p* value is significant < 0.05.

*ND* not detected.

Statistical analysis revealed no significant differences for the individual genes *bla*_TEM_ (X^2^ = 3.644, *P* = 0.302), *bla*_SHV_ (X^2^ = 1.296, *P* = 0.861), *bla*_CTX-M_ (X^2^ = 1.241, *P* = 0.619), *Uge* (X^2^ = 1.194, *P* = 0.769), *mrkD* (X^2^ = 1.098, *P* = 0.7775), and *luxS* (X^2^ = 1.458, *P* = 0.691).

Table 3 presents the pattern of biomarker resistance genes and biofilm-associated genes in selected ESBL-producing *Klebsiella pneumoniae* isolates harboring biofilm-associated genes. Isolates from poultry sources exhibited notable diversity, with two carrying the complete set of resistance genes (*bla*_TEM_*, bla*_SHV_*, bla*_CTX-M_*, bla*_OXA-1_) and biofilm-associated genes (*Uge, mrkD, luxS*). Similarly, equine sources contributed four isolates with a comparable profile, all possessing *bla*_TEM_*, bla*_SHV_*, bla*_CTX-M_, and the biofilm-associated genes *Uge, mrkD,* and *luxS.*

**Table 3:** The pattern of biomarker resistance genes and biofilm-associated genes in selected ESBL-producing *Klebsiella pneumoniae* isolates harboring biofilm-associated genes, used for antibiotic susceptibility testing and experimental analysis.

Source	Sample	No. of isolates	Biomarker resistance genes	Biofilm genes
Poultry Farm	Poultry	2	*bla*_TEM_ *, bla*_SHV_ *, bla*_CTX-M_ *, bla*_OXA-1_	*Uge, mrkD,* luxS
	Poultry	4	*bla*_TEM_ *, bla*_SHV_ *, bla*_CTX-M_	*Uge, mrkD,* luxS
Equine Farm	Equine	4	*bla*_TEM_ *, bla*_SHV_ *, bla*_CTX-M_	*Uge, mrkD,* luxS
Equine Farm	Wild Birds (Crow)	1	*bla*_TEM_ *, bla*_SHV_ *, bla*_CTX-M_ *, bla*_OXA-1_	*Uge, mrkD,* luxS
Poultry Farm	Wild Birds (Crow, Pigeon)	2	*bla*_TEM_ *, bla*_SHV_ *, bla*_CTX-M_	*Uge, mrkD,* luxS
Equine Farm	Water	1	*bla*_TEM_ *, bla*_SHV_ *, bla*_CTX-M_	*Uge, mrkD,* luxS
Poultry Farm	Water	2	*bla*_TEM_ *, bla*_SHV_ *, bla*_CTX-M_	*Uge, mrkD,* luxS
Poultry Farm	Human	1	*bla*_TEM_ *, bla*_SHV_ *, bla*_CTX-M_	*Uge*
Equine Farm	Human	1	*bl*a_TEM_*, bla*_SHV_	*Uge*
Total		18		

**Fig. 1:**
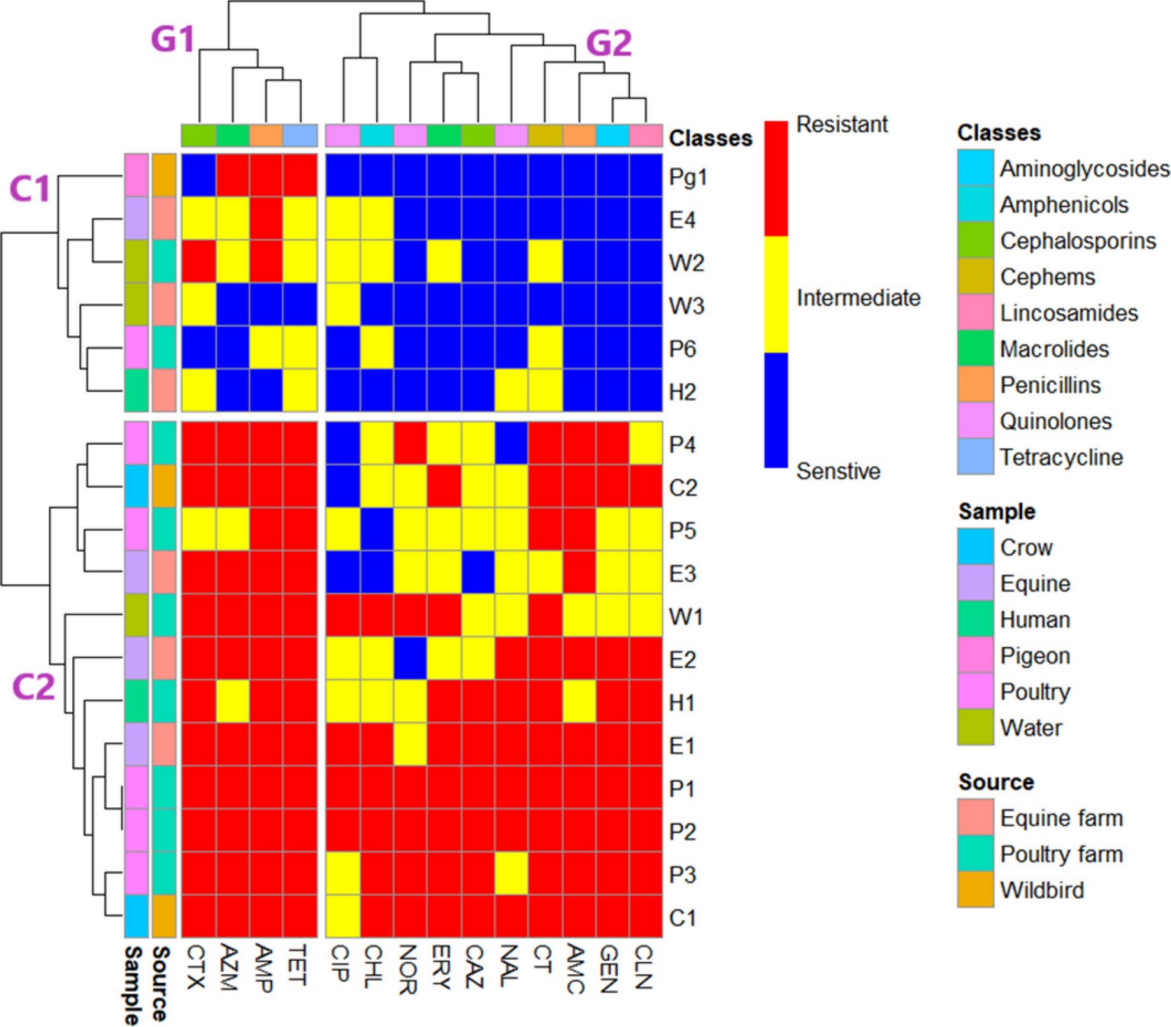
A heatmap with hierarchical clustering was generated using R packages to visualize ESBL-producing *Klebsiella pneumoniae* isolates carrying biofilm-associated genes. The isolates were grouped into clusters C1 and C2 based on their phenotypic resistance profiles, while the top of the heatmap (G1 and G2) illustrates the patterns of antibiotic resistance.

Wild bird isolates were diverse, including one isolate containing the full set of resistance and biofilm genes, which was identified on an equine farm. Water samples, however, displayed consistency, with all three isolates from equine and poultry farms harboring *bla*_TEM_*, bla*_SHV_*, bla*_CTX-M_, and the biofilm-associated genes *Uge, mrkD*, and *luxS.* Human-derived isolates were variable, with one isolate carrying only *bla*_TEM_*, bla*_SHV_, *bla*_CTX-M_, and *Uge*, while another had *bla*_TEM_*, bla*_SHV_*,* and *Uge*. The antibiotic resistance profiles of selected isolates recovered from Poultry, Equine, Human, and Environmental Samples, across different antibiotic classes, were displayed in Table 4 and Fig. 1.

**Table 4:** Antibiotic resistance profiles of selected isolates recovered from Poultry, Equine, Human, and Environmental Samples.

Class	Antibiotics*	No. of resistant *K. pneumoniae* isolates (%)					
		Poultry n = 6 (%)	Equine n = 4 (%)	Water n = 3(%)	Wild Birds n = 3 (%)	Human n = 2(%)	Total n = 18 (%)
Aminoglycosides	GEN	4 (66.6)	2 (50.0)	0 (0.0)	2 (66.6)	1 (50)	9 (50.0)
Penicillins	AMP	5 (83.8)	4 (100)	2 (66.6)	3 (100)	1 (50)	15 (83.3)
	AMC	5 (83.8)	3 (75.0)	0 (0.0)	2 (66.6)	0 (0)	10 (55.5)
Macrolides	ERY	3 (50.0)	1 (25.0)	1 (33.3)	2 (66.6)	1 (50)	8 (44.4)
	AZM	4 (66.6)	3 (75.0)	1 (33.3)	3 (100)	0 (0)	11 (61.1)
Cephalosporins	CTX	4 (66.6)	3 (75.0)	2 (66.6)	2 (66.6)	1 (50)	12 (66.6)
	CAZ	3 (50.0)	1 (25.0)	0 (0.0)	1 (25.0)	1 (50)	6 (33.3)
Quinolones	NAL	2 (33.3)	1 (25.0)	0 (0.0)	1 (33.3)	1 (50)	5 (27.7)
	CIP	2 (33.3)	1 (25.0)	1 (33.3)	0 (0.0)	0 (0)	4 (22.2)
	NOR	4 (66.6)	0 (0.0)	1 (33.3)	1 (33.3)	0 (0)	6 (33.3)
Amphenicols	CHL	3 (50.0)	1 (25.0)	1 (25.0)	1 (25.0)	0 (0)	6 (33.3)
Tetracycline	TET	5 (83.8)	3 (75.0)	1 (33.3)	3 (100)	1 (50)	13 (72.2)
Cephems	CT	5 (83.8)	2 (50.0)	1 (33.3)	2 (66.6)	1 (50)	11 (61.1)
Lincosamides	CLN	3 (50.0)	2 (50.0)	0 (0.0)	2 (66.6)	1 (50)	8 (44.4)

*GEN* gentamycin, *AMP* ampicillin, *AMC* amoxicillin/clavulanic acid, *ERY* erythromycin, *AZM* azithroyn, *CTX* cefotaxime, *CAZ* ceftazidime, *NAL* nalidixic acid, *CIP* ciprofloxacin, *NOR* norfloxacin, *CHL* chloramphenicol, *TET* tetracycline, *CT* ceftazidime, *CLN* clindamycin.

### The effects of different disinfectants on ESBL-producing *Klebsiella pneumoniae* isolates harboring biofilm-associated genes

**Fig. 2:**
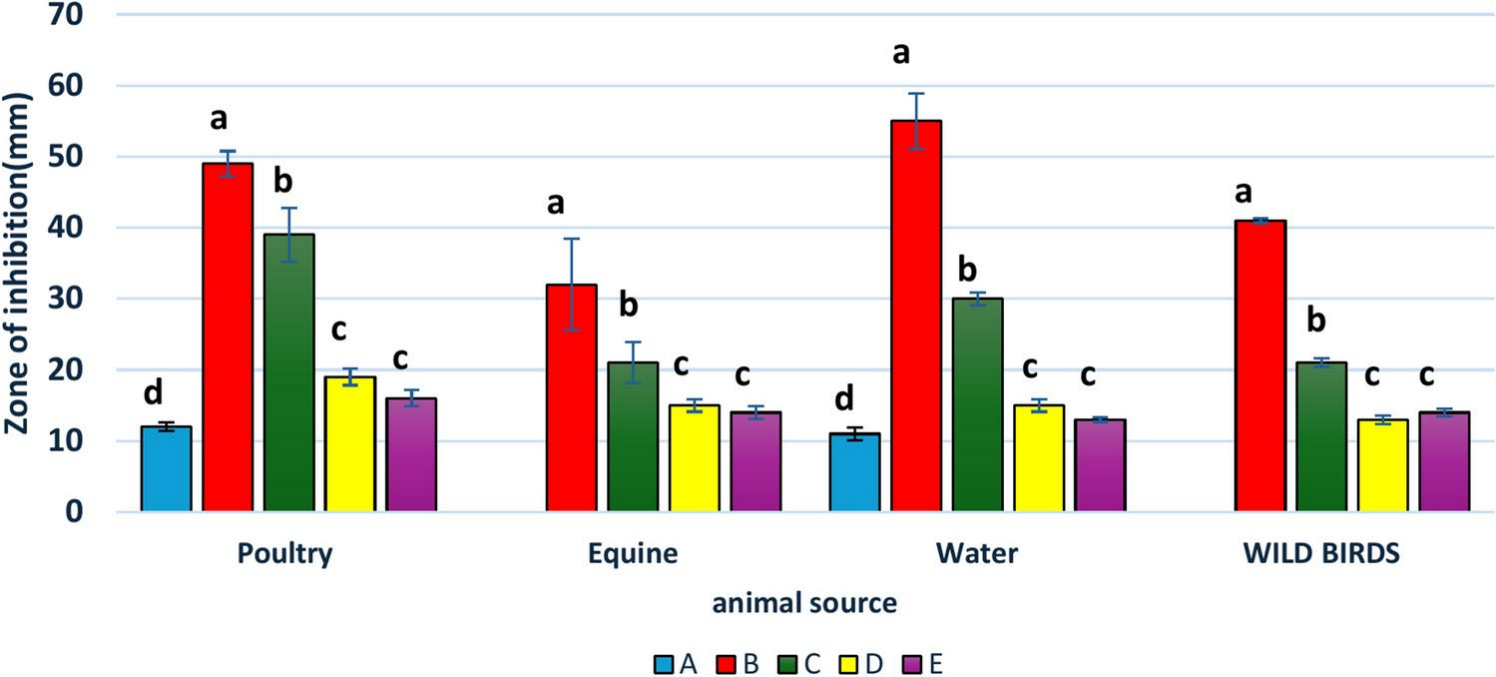
In vitro effectiveness of five disinfectants (at 100% concentration) against ESBL-resistant *Klebsiella pneumoniae* isolates carrying biofilm-associated genes, sourced from various animal and environmental samples. ^a,b,c,d^means significant differences between strains.

Table 5 shows the sizes of the inhibition zones generated by the different disinfectants. The product based on sodium hypochlorite and containing peracetic acid and hydrogen peroxide was significantly more active (p < 0.05) on all *K. pneumoniae* strains. We observed no significant differences between the different strains of *K. pneumoniae* when using disinfectant E (p > 0.05). On the other hand, the results in Fig. 2 show that among different *K. pneumoniae* isolates, poultry and water had the highest readings for the inhibition zone. In contrast, diseased equine had the lowest inhibition zone. In addition, disinfectant B, followed by disinfectant C, had the highest reading for the inhibition zone. Disinfectants based on peracetic acid and hydrogen peroxide were much more effective against *K. pneumoniae* isolates than disinfectant (A), which had the lowest inhibition zone (p < 0.001).

**Table 5:** The median and interquartile range (IQR) of the inhibition zones (mm) produced by the disinfection products against selected *K. pneumoniae* isolates.

Sampling	Origin	Inhibition zone (mm) products tested				
		Disinfectant A	Disinfectant B	Disinfectant C	Disinfectant D	Disinfectant E
Poultry	Diseased (n = 6)	12^a^ IQR 11–13	49^ab^ IQR 47–53	40^a^ IQR 32–45	19^a^ IQR 17–21	16 IQR 14–18
Equine	Diseased (n = 4)	0^b^	32^b^ IQR 28–49	21^b^ IQR 16–26	15^b^ IQR13-16	14 IQR13-16
Water	Environmentaln(n = 3)	11^a^ IQR 10–13	55^a^ IQR 52–65	30^ab^ IQR 28–31	15^ab^ IQR 14–17	13 IQR 13–14
Wild	Environmental (n = 3)	0^b^	41^ab^ IQR 40–42	21^b^ IQR 20–22	13^b^ IQR 12–14	14 IQR 13–15
*P* value		0.000	0.02	0.002	0.009	0.191

Disinfectants used: Disinfectant A (Potassium peroxymonosulfate,1%), Disinfectant B (peracetic acid and hydrogen peroxide, 1%), Disinfectant C (17.2% isopropanol), Disinfectant D (quaternary ammonium compounds, 0.5%), and Disinfectant E (quaternary ammonium compounds & glutaraldehyde, formaldehyde, 1%).

Different zones of different superscript letters (^a,b,c & ab^) of the same column are significantly different at P ≤ 0.05.

## Discussion

*Klebsiella pneumoniae* is recognized as a significant pathogen in both nosocomial and community-acquired infections^45^. While it typically resides in the respiratory tract and intestines of healthy individuals, it has the potential to cause severe infections in various tissues and organs, including pneumonia, meningitis, liver abscesses, urinary infections, and sepsis^46^. This study highlights the prevalence of *K. pneumoniae* in both animals and environmental samples, demonstrating its widespread presence. *K. pneumoniae* was detected in poultry, equine, and wild bird samples, with a notable presence in avian species. It was also identified in environmental sources like water and feed and human hand swabs. Given its ubiquity and pathogenic potential in humans and animals, *K. pneumoniae* has attracted significant global attention from researchers^1^. The pathogen accounts for approximately 14–20% of infections in the respiratory tract, biliary duct, surgical wounds, and urinary tract^47^. These findings underscore the importance of monitoring and controlling *K. pneumoniae* across various environments to mitigate its impact on public and animal health.

*Klebsiella pneumoniae* is well-known for its ability to form biofilms, a critical virulence factor that enhances its antibiotic resistance and shields it from immune responses, thereby increasing its persistence in clinical environments^48,49^. Biofilms are composed of a matrix of polysaccharides, extracellular DNA, and proteins, that protect the bacteria and limit antibiotic penetration, contributing to its resistance^15,50^. Most *Klebsiella pneumoniae* isolates exhibit strong biofilm-forming capabilities, which are closely associated with extensive virulence and antimicrobial resistance genes^51^.

In this study, all *Klebsiella pneumoniae* isolates tested positive for the *uge* gene. Specifically, isolates came from poultry, equines, wild birds, water, feed, and human samples. In comparison, most isolates were positive for the *mrkD* gene and the *luxS* gene. These results are consistent with findings from Arafa and Kandil^22^, who reported that *K. pneumoniae* isolates from equines were positive for *uge* and *luxS*. Similarly, Hamam et al.^52^ found that *K. pneumoniae* isolates from urinary tract infections carried *uge* gene.

Studies from different regions reveal varying gene prevalence. Elmeslemany and Yonis^53^ identified the *uge* gene in ducklings. In Brazil, Davies et al.^54^ detected both the *uge* and *mrkD* genes in wild bird isolates. Similarly, Daehre et al.^55^ in Germany and Ferreira et al.^56^ in Brazil found widespread *mrkD* gene in broiler chickens and ICU isolates. In Malaysia, Barati et al.^57^ found the *uge* gene in water isolates.

Conversely, Ashwath et al.^19^ observed a reduction in *mrkA* and *mrkD* gene expression in *K. pneumoniae* from clinical specimens, with expression levels varying among strong and moderate biofilm formers. In Iraq, Muhsin et al.^58^ noted the presence of the *mrkD* gene in clinical and environmental isolates, indicating regional differences in gene prevalence.

In recent years, with the wide application of antibiotics, the resistance of clinically isolated *K. pneumoniae* has become stronger and stronger. *K. pneumoniae* that produce extended-spectrum beta-lactamase (ESBLs) have been observed and introduced significant challenges to antibiosis^59^.

All *Klebsiella pneumoniae* isolates tested positive for the *bla*_TEM_ and *bla*_SHV_ genes, with most also harboring the *bla*_CTX-M_ gene. The *bla*_OXA-1_ gene was less commonly detected, found in a few isolates from poultry and wild birds. These findings are consistent with those of Arafa et al.^5^, who reported the presence of the *bla*_TEM_ gene in equine isolates in Egypt, while *bla*_SHV_ and *bla*_CTX-M_ genes were not detected. Regional variations in the prevalence of resistance genes have been noted. For example, Siqueira et al.^60^ characterized antimicrobial resistance in K. pneumoniae isolates from dogs and a horse in Brazil, identifying multiple ESBL genes, including *bla*_CTX-M_, *bla*_TEM_, and *bla*_SHV._.

The detection of *bla*_TEM_ and *bla*_SHV_ genes in *K. pneumoniae* isolates from different sources, including animals and the environment, underscores the significant role of these genes in antimicrobial resistance (AMR). Recent research highlights the increasing concern over the transmission of antibiotic-resistant genes between domestic animals and their owners, emphasizing the zoonotic potential of resistant bacteria^61,62^. This underscores the need for vigilant monitoring and control measures to manage the spread of antibiotic resistance.

As observed in this study, the coexistence of multiple ESBL genes with biofilm genes within the same strain indicates a significant potential for gene transfer, which plays a role in the spread of antimicrobial resistance (AMR). This observation is further supported by the findings of Elmeslemany and Yonis^53^ and other researchers, who emphasized the link between integrons and ESBL genes, suggesting that these genes may be located on the same plasmid.

The increased prevalence of multidrug-resistant pathogens poses significant challenges in treating bacterial infections, largely attributed to the misuse of antimicrobials in both healthcare settings and communities. These findings highlight the need for coordinated efforts to address AMR across different sectors.

AMR is a critical One Health issue, with the spread of multidrug-resistant pathogens among animals, humans, and the environment complicating infection treatment due to biofilms. This underscores the need for vigilant monitoring and control measures to manage the spread of antibiotic resistance.

Chemical biocides are widely used to control infections in healthcare, industry, and targeted home hygiene, interacting with bacterial cells primarily by targeting the cytoplasmic membrane and enzymes. However, improper usage or sublethal concentrations of biocides can lead to antimicrobial resistance and promote the transfer of resistance genes^63^. This concern is heightened by the potentially harmful effects of chronic biocide exposure on human health and the selection of less susceptible bacterial isolates^64^.

Quaternary ammonium compounds (QACs), such as benzalkonium chloride, are commonly used in disinfectants for medical and food-processing environments, as well as in cosmetics and pharmaceuticals^65^. In our in vitro study, disinfectants containing peracetic acid, isopropanol, and QACs were the most effective against bacterial species, with QAC-based disinfectants showing particular efficacy in Bangladesh. However, prolonged use of QACs in Egypt has led to resistance in poultry settings^66^.

Sodium hypochlorite has been shown to reduce biofilm activity in bacteria like *Enterococcus faecalis* and *Klebsiella pneumoniae*, improving effectiveness at higher concentrations^67,68^. Recent studies suggest that combining oxidizing agents like sodium hypochlorite and hydrogen peroxide with novel agents, such as chlorhexidine-conjugated gold nanoparticles, can be particularly effective in eliminating *K. pneumoniae* and preventing biofilm formation^69,70^. While our study found that disinfectant A had minimal effectiveness, sodium hypochlorite-based disinfectants have been reported to be highly effective against certain bacterial species, such as *S. lentus* and *M. luteus*^42^. Implementing a sustainable control strategy and raising breeders’ knowledge and awareness is essential for the efficient management and mitigation of *K. pneumoniae* infection.

## Conclusion

*Klebsiella pneumoniae* is notably present in poultry, resident birds, water, equine populations, and humans, with biofilm formation and ESBL genes contributing to its persistence. This investigation revealed that sodium hypochlorite product inhibits and clears *K. pneumoniae* with varying antibiotic resistance. The effect increased as the concentration increased within the range of bactericidal concentration. Comprehensive surveillance, biosecurity protocols, and efficient infection control methods are essential to minimize its spread and lower the risk of infection in animal and human populations. Collaborative efforts among veterinarians, wildlife biologists, public health officials, and environmental scientists are essential to understanding and addressing the complex dynamics of *K. pneumoniae* transmission across these interconnected ecosystems.

## Supplementary Information

Supplementary Information.

## Data Availability

All the data generated or analyzed in this study are included in this published article.
