# Supplementary material for: Emerging biofilm formation and disinfectant susceptibility of ESBL-producing Klebsiella pneumoniae
Source: Sci Rep. 2025 Jan 10;15:1599. doi: 10.1038/s41598-024-84149-x (PMC11724021; doi:10.1038/s41598-024-84149-x)
Supplement: Supplementary file 1 — Supplementary Information. [file 41598_2024_84149_MOESM1_ESM.docx]

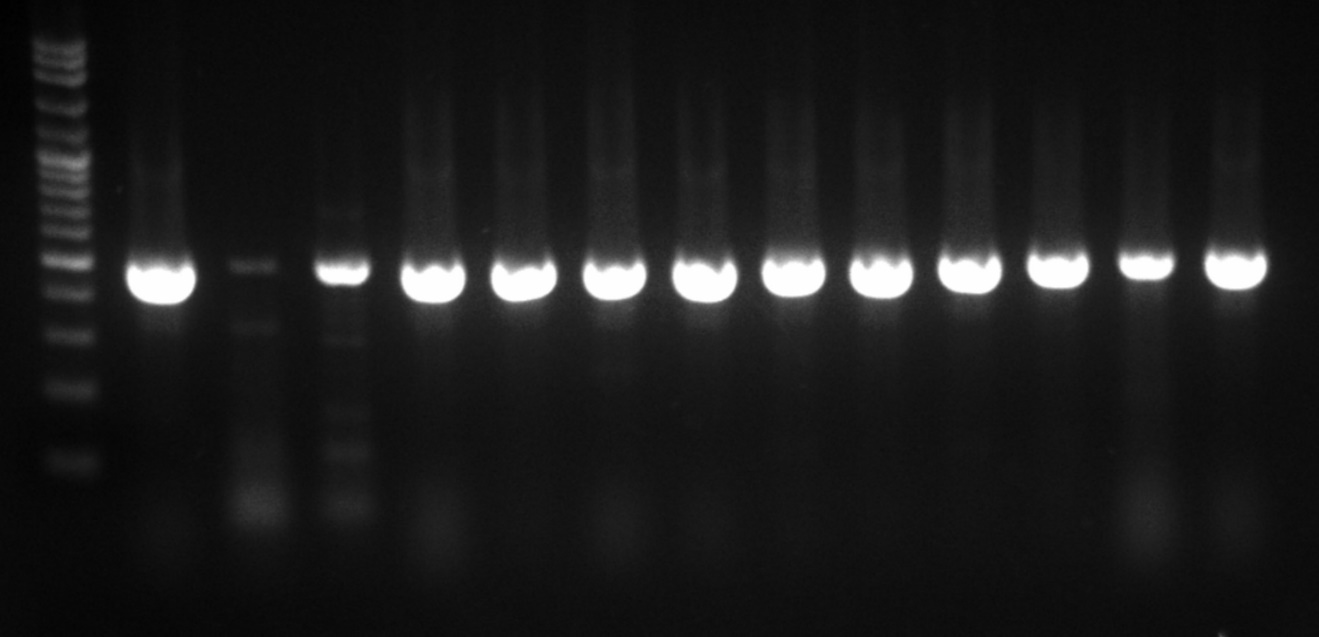


**Fig.1 Agarose gel electrophoresis of PCR product amplified from *K. pneumoniae* *luxS* gene (447 bp). Lanes 1, 100 bp DNA Ladder; Lanes 2-14, positive *K. pneumoniae* isolates.**

**
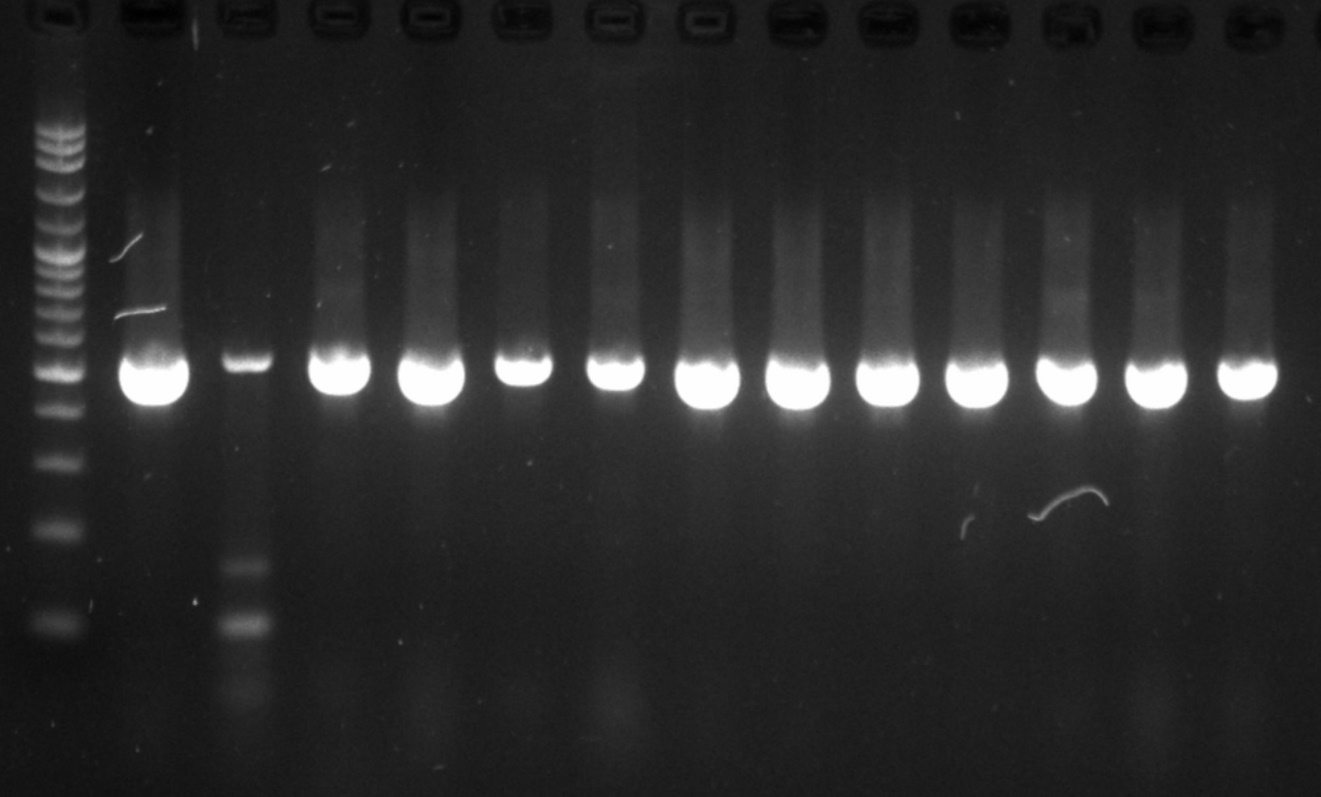
**

**Fig.2 Agarose gel electrophoresis of PCR product amplified from *K. pneumoniae* *uge* gene (535 bp). Lanes 1, 100 bp DNA Ladder; Lanes 2-14, positive *K. pneumoniae* isolates.**

**
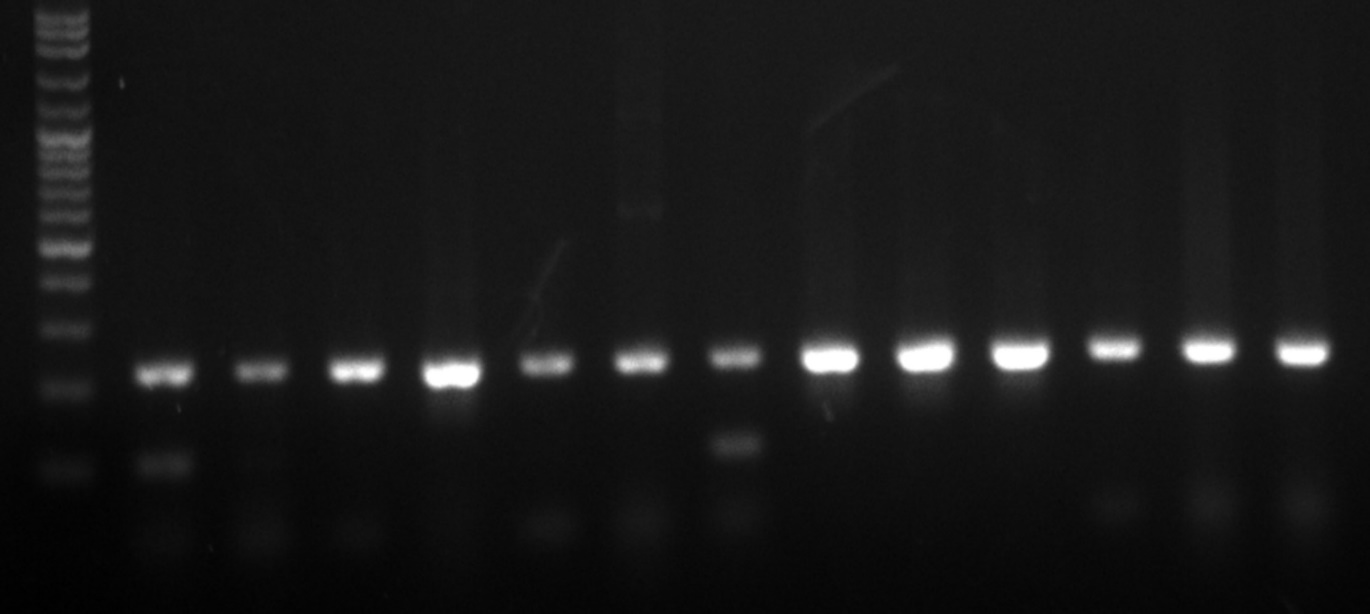
**

**Fig.3 Agarose gel electrophoresis of PCR product amplified from *K. pneumoniae* *mrkD* gene (226 bp). Lanes 1, 100 bp DNA Ladder; Lanes 2-14, positive *K. pneumoniae* isolates.**
